# Task analysis method for procedural training curriculum development

**DOI:** 10.1007/s40037-013-0100-1

**Published:** 2013-12-24

**Authors:** Jakeb D. Riggle, Michael C. Wadman, Bernadette McCrory, Bethany R. Lowndes, Elizabeth A. Heald, Patricia K. Carstens, M. Susan Hallbeck

**Affiliations:** 1 University of Nebraska – Lincoln, W342 Nebraska Hall, Lincoln, NE 68588 USA; 2 University of Nebraska Medical Center, Omaha, NE USA; 3 Mayo Clinic, Rochester, MN USA

**Keywords:** Cognitive task analysis, Task analysis, Training, Procedures, Central venous catheter (CVC)

## Abstract

A central venous catheter (CVC) is an important medical tool used in critical care and emergent situations. Integral to proper care in many circumstances, insertion of a CVC introduces the risk of central line-associated blood stream infections and mechanical adverse events; proper training is important for safe CVC insertion. Cognitive task analysis (CTA) methods have been successfully implemented in the medical field to improve the training of postgraduate medical trainees, but can be very time-consuming to complete and require a significant time commitment from many subject matter experts (SMEs). Many medical procedures such as CVC insertion are linear processes with well-documented procedural steps. These linear procedures may not require a traditional CTA to gather the information necessary to create a training curriculum. Accordingly, a novel, streamlined CTA method designed primarily to collect cognitive cues for linear procedures was developed to be used by medical professionals with minimal CTA training. This new CTA methodology required fewer trained personnel, fewer interview sessions, and less time commitment from SMEs than a traditional CTA. Based on this study, a streamlined CTA methodology can be used to efficiently gather cognitive information on linear medical procedures for the creation of resident training curricula and procedural skills assessments.

## Introduction

A central venous catheter (CVC) is an important and potentially life-saving medical device used to draw blood and administer fluids and medications. CVCs are most commonly used in critical care and emergency situations. An estimated 3 million central lines are inserted in the US each year [1]. Although essential to the delivery of care, CVCs can cause a number of insertion-related complications.

Insertion of a CVC can cause a number of mechanical complications including damage to the blood vessels, heart, or lungs. Additionally, and perhaps more critically, the catheter provides a direct route to the blood stream for microorganisms, potentially leading to a central line-associated blood stream infection (CLABSI). Approximately 3.9 % of CVC insertions result in a mechanical adverse event [2], and it is estimated that each year up to 80,000 CLABSIs occur resulting in nearly 28,000 deaths [3, 4]. The abundant use of CVCs and their susceptibility to life-threatening adverse events led the Joint Commission to institute a National Patient Safety Goal for 2010, 2011, and 2012 to prevent CLABSIs [5].

As a result of the Joint Commission directive and other efforts, a substantial amount of research has been conducted to improve the procedural steps, training, and techniques used when inserting a CVC or teaching the procedure [4, 6–10]. As expected, the number of complications caused by CVC insertion decreases as physician experience increases [11, 12]. Patient simulators offer a platform for procedural practice and can improve procedural competency and decrease complication rates [6, 7, 13]. Similarly, researchers have used task analysis techniques to develop more comprehensive tools for medical procedural training and evaluation with and without simulation [9, 14–18].

A number of published studies report comprehensive task lists for the CVC procedure [13, 14, 19]. However, it is critical that postgraduate medical trainees (residents or house officers) also understand the purpose of the procedure, potential problems they might encounter, and alternative actions they may need to take while performing the procedure. With experience and practice, expert practitioners (i.e. attending physicians) begin to perform procedures in an automated (subconscious) manner. Thus, while instructing trainees, they may omit 50 % or more of the steps and cognitive decision points necessary to correctly perform the procedure [18, 20]. Cognitive task analysis (CTA) methods were developed to elucidate these types of cognitive cues to describe the thought processes involved in complex decision-making. CTA uses cognitive psychology and cognitive engineering principles to deconstruct the automated skills of experts [17], allowing these unseen skills to be passed on to trainees.

CTA has been applied within the health care field before, particularly for creating skills assessment checklists for procedures such as a tracheostomy, laparoscopic cholecystectomy, and CVC insertion [8, 9, 17, 21]. These skills assessment tests proved to be useful and accurate in determining the skill level of newly trained residents and experienced physicians alike [8, 12], yet the CTA data have rarely been used to develop new curricula for teaching the procedures [15, 17, 21]. Most published research that used CTA to create a medical procedure curriculum does not publish the cognitive aspects of the procedure, making it difficult to adapt or generalize the findings of these works [9, 14].

A variety of CTA methods have been proposed to gather cognitive decision points from subject matter experts (SMEs) [22–24]. A typical CTA begins with multiple in-depth semi-structured interviews with SMEs on the task being studied, followed by data review and analysis, and completed with a validation of the final procedural and cognitive cues lists by another set of SMEs. A CTA is a thorough but time-consuming task for researchers and SMEs and can require many hours of interviews, transcription, and analysis [20, 22–25]. Most CTA methods are designed to be applied to branching tasks, where a decision based on input at one point can lead to a number of different actions, outcomes, and new decisions. Many CTA methods are unnecessarily complex for medical procedures, such as CVC insertion. Developing a resident procedural training curriculum would require conducting a CTA for every medical procedure, which would be time prohibitive for SMEs. Therefore, a minimally time-intensive task-analysis method was sought.

The PARI method of conducting a CTA was modified for linear medical procedures to enable physicians to quickly develop a thorough and in-depth curriculum for training first-year residents. The PARI method is a more structured and scripted format of CTA described by Hall et al. [25]. PARI is an acronym for precursor, action, result, and interpretation. The PARI interview methodology involves a video-recorded response to a proposed problem by an SME (i.e. a hypovolemic patient requires central venous access for treatment). The interviewer then reviews the video with the SME and breaks the response into actions performed, and probes the SME for the information and knowledge that lead to each individual action (i.e. the precursor to the action). The SME is then asked about common and possible outcomes from that action (i.e. results of the action), and how each result should be acted upon or interpreted. These probes are asked of each action related by the SME during five ‘rehash sessions.’ The final step of the interview process asks the SME to group and reorder the actions to follow their mental model of the solution. This interview process should then be repeated with multiple SMEs addressing similarly proposed problems. Information from these interviews is then abstracted and analyzed across SMEs to create a thorough list of actions and alternate actions (i.e. a procedural list) and a list of cognitive cues and interpretations associated with each action (i.e. a cognitive list). This combined list is then discussed, modified, and validated by a group of SMEs and is then used to evaluate the decision-making skills of novices [25].

The linear format of many medical procedures limits the amount of probing necessary to gain a clear picture of the important steps and cognitive cues encountered during the procedure. Most steps have only a few alternative actions, leading to a minimally branched task list. Specifically, if the outcome of an action is not appropriate or acceptable, the same action is attempted again. This allows for a more straightforward interview process that could be reduced from an interview format to simpler forms of gathering information.

The target user of the task list created through this method is the medical professional creating a procedure training programme. As such, the user is already an expert in the procedure, but may need assistance delineating distinct steps and important decision points to base a curriculum on [18]. A full CTA would provide all information necessary to perform the procedure; because the target user is already an expert in the procedure, only the high-level cognitive decision points need to be elucidated.

## Methods

A step-by-step procedural training task list and accompanying cognitive probe list were created using the information from one CTA and simulation-based interview and a survey amongst SMEs. The combined list was verified by three SMEs while performing a simulated CVC procedure. The study procedures were granted exempt status by the hospital’s institutional review board, and all subjects provided written informed consent.

### Participants

To be considered an SME, participants must have performed more than 20 CVCs on patients, the guideline suggested by the Residency Review Committee for Emergency Medicine of the Accreditation Council for Graduate Medical Education to demonstrate procedural competency [26]. Three or four SMEs has been shown to be the number of experts necessary to capture the optimum amount of information for the CVC procedure using a CTA methodology [9]. One SME in CVC insertion was recruited from the emergency medicine department at a local academic medical centre to complete a CTA and simulation-based interview. Fifty-two additional potential SMEs were recruited from the anaesthesiology, internal medicine, and emergency medicine departments to complete a PARI-based survey, with 13 complete surveys returned by respondents who qualified as an SME (response rate of 25 %). Of the completed surveys, 54 % were from the internal medicine department, 23 % from the anaesthesiology department, and 23 % from the emergency medicine department. Three of the completed surveys were from residents (postgraduate years 2–4), 5 were from fellows (postgraduate year 5), and 5 were from faculty members. Survey participants ranged in age from 29 to 53 years, with a median age of 33 and a mean of 35 years. Most respondents were male (92 %).

Three additional SMEs were recruited to complete the verification portion of the study. Two of these SMEs were from internal medicine: one was a fellow and one was a faculty member. The third SME was a faculty member in the nephrology department.

### Apparatus

The simulator used in this study was a partial-task central line simulation mannequin (Central Venous Access Head Neck & Upper Torso Ultrasound Training Model, Model Number BPH600; Blue Phantom, Redmond, WA) with a bedside ultrasound machine and triple-lumen central line kit. Participants were instructed to announce the use of unavailable items (i.e. sterile gowns) during the simulated procedure.

The simulated CVC procedure and interview were performed in a clinical skills simulation lab and recorded using ceiling-mounted video cameras.

### Procedure

#### Procedure simulation and interview

The SME was instructed to insert a CVC via the internal jugular approach with ultrasound guidance using the patient simulator. The mannequin was set up to simulate a hypovolaemic patient, which is generally recognized as a difficult procedural case. The participant was asked to explain the procedure as if teaching it to a first-year resident.

Following the simulated procedure, the SME was asked to verbally review the steps necessary to perform a CVC, including steps they may have left out earlier. The steps described in the simulated procedure and in the verbal recall were transcribed into a ‘procedures’ list that was pre-populated using published literature [10, 13, 14, 19, 27]. The SME then participated in a recorded CTA-based interview conducted by the researcher, wherein the video of his CVC procedure was reviewed. The interviewer confirmed all steps of the procedure with the SME. The video was paused at any steps on the procedures list, and the researcher asked the participant a series of questions developed using the PARI CTA interview methodology (Table 1) [25]. These questions were directed at determining the steps necessary to perform a CVC insertion, alternative outcomes, optional steps, and the information required to make decisions when unexpected outcomes are encountered. Lastly, the SME was asked to order and group the procedure steps as he saw fit. The video of the interview was then transcribed and reviewed to ensure all information was recorded accurately.

**Table 1:** CTA interview and survey questions

Interview	Survey
1. ‘At this point in the procedure, which is *localizing anatomy and target vessel with ultrasound* what you are doing now? What information led you to perform this action?’	1. Is this step in the correct position in the CVC procedure sequence? (Yes/No) If No, which step should it follow?
2. ‘What happened as a result of this action? If you were teaching the procedure to a novice, what would you tell them *could* happen as a result of this action? What would these alternative outcomes mean?’	3. What is the normal outcome of this step? 4. What are other potential outcomes of this step?
3. ‘Are there other actions you could have performed at this point in the procedure instead? Compare each alternative to the original action and explain why one action is preferred over the other.’	Blank ‘step sections’ were included at the end of the survey for the addition of steps that may have been left out of the original format
4. ‘What other information would have been helpful or useful when considering this action (*localizing anatomy and target vessel with ultrasound*)? Compare each piece of information to the original information used and explain why one is preferred’	2. What information is needed to perform this step correctly?

Questions are numbered as they appear in the interview and survey. Rows are aligned to match the process within the modified PARI methodology

*1* Action and precursors, *2* Results and interpretation, *3* Alternative actions, *4* Information to perform the action

#### Development and distribution of task analysis survey

To reduce the amount of time required for additional SME interviews, a PARI-based survey was developed using information from the simulation interview and a focused literature search for procedural guides founded using task analysis and cognitive task analysis techniques [10, 13, 14, 19, 27]. One anaesthesiology and two internal medicine faculty physicians provided feedback on the format and method of distribution of the survey and assisted in distribution. The survey was distributed to 52 residents, fellows, and attending physicians in the internal medicine, emergency medicine, and anaesthesiology specialities who regularly insert CVCs.

Survey participants were given a list of steps necessary for inserting a CVC created using literature and the information gathered from the simulation portion of the study. The majority of the survey consisted of a set of task analysis questions for each step involved in performing a CVC similar to those asked in the task analysis interview (Table 1). The survey also allowed participants to add any additional steps that were not listed. Finally, the survey asked participants to order the steps and group them as they saw fit. CVC procedural information gleaned from the survey process and simulation interview were then converted into procedure task and cognitive probe lists.

#### Data analysis

Survey responses were grouped by actions and examined for prevalence and trends, then converted to cognitive probes beginning with ‘Identify,’ ‘Determine,’ or ‘Consider,’ as per Sullivan et al. [18]. The order of actions for the procedure list was determined using the mean task sequence number from survey responses and ‘ordered functions’ (i.e. if one task could only be performed following another task). The procedure task and cognitive probe lists were then grouped by tasks following the approximate groups created by the SMEs. This combined procedural task and cognitive probe list was then presented to SMEs for verification using a simulated procedure.

#### Simulation-based verification

Three SMEs were asked to review the previously compiled procedural task and cognitive probe list and note any changes they deemed necessary. They were then instructed to perform a CVC using the internal jugular approach with ultrasound guidance on the simulation mannequin. The mannequin was set up to simulate a normovolaemic patient. Similar to the simulation portion of the study, the participants were asked to treat the mannequin as if it were a patient, and to explain the procedure as they performed it. Any differences in procedure were recorded on the task list by the researcher and reviewed with the SME when the simulation was complete.

Following the simulated procedure, the SMEs were asked to review the revised procedural and cognitive task. They were asked to make any final changes and add comments to the task list. These changes were then compiled by the researchers to develop the final procedural task and cognitive probe list. As the changes made during the verification phase were minimal and conserved amongst all three SMEs, it was deemed unnecessary to conduct a second round.

#### Time evaluation

Time is an important commodity in the health care field, and blocks of available time for physicians to participate in interviews as SMEs is limited. Based on the time-reduction goal for this project, time spent in task analysis simulations, interviews, and discussions by SMEs was logged for the simulation/interview and verification stages of the methodology.

## Results

A step-by-step procedural task list and accompanying cognitive probe list (Table 2) were created using the information from one CTA-based interview and 13 survey respondents (see Table 3 for demographic information). The combined list was verified by three SMEs while performing a simulated CVC procedure. This verified checklist for the internal jugular approach using ultrasound guidance includes at least six more procedural steps (29 total) than what is found in published literature [10, 13, 14, 19, 27] and a total of 39 cognitive decision points for training. Initial procedural steps and cognitive decision points were created using published literature and the simulation task analysis interview. No new procedural steps were added via survey responses, but the majority of the cognitive decision points were gathered from this phase of data collection. The verification portion of the study ensured the checklist was accurate to a simulated procedure. Verification resulted in a minor reorganization of the steps (moving ‘Remove patient from Trendelenburg position’ from before ‘Secure catheter with suture/staples’ to after ‘Place sterile dressing’) to increase patient comfort by decreasing the apparent procedure length.

**Table 2:** Procedural tasks and cognitive probes identified via CTA

Procedural tasks	Cognitive probes	Group
Informed consent/TJC timeout	Identify risks, complications, and benefits of procedure	Setup/procedure preparation
Place patient in Trendelenburg position	Determine if patient can tolerate position	
Inspect anatomy with ultrasound (US) and choose CVC site	Identify anatomical landmarks and consider alternate sites	
**Check for contraindications with US**	Identify problems with patient status or anatomy and consider alternate sites	
**Open all kits on stand**	Determine proper location for stand and kits and identify all equipment in kits	
Disinfect chosen CVC site with chlorhexidine	Consider disinfecting multiple sites simultaneously	
**Position machinery**	Determine proper location for machinery	
Wash hands		Sterile practice
Sterile gown, gloves, hat, and mask	Identify breaks in sterile practice	
Full body sterile drape on patient	Identify breaks in sterile practice	
Sterile sheath on ultrasound probe	Identify breaks in sterility of probe sheath and sterile attire	
**Lay out equipment**	Determine proper location for equipment and identify presence of all equipment	Equipment preparation
Remove caps from all catheter ports, flush with saline	Determine patency of all catheter lumens and identify entrapped air in catheter	
Localize anatomy and target vessel with US	Identify important anatomical landmarks on skin and US	Vessel access
Anaesthetize skin	Consider anaesthetizing deep structures	
Cannulate vein with needle while aspirating, using US guidance	Determine needle angle to use, identify venous blood return, and identify improper cannulation	
Remove syringe from needle (if not using pass-through needle)	Determine needle remains in vessel and identify venous blood return	
Advance guide wire through needle	Determine direction to advance the wire and consider depth to advance wire	
Remove introducer needle	Determine that guide wire is secure at all times	
**Verify position of wire in vein using US**	Identify wire in correct vessel and correct direction	
Cut skin with scalpel	Consider proper depth to cut to avoid complications	Catheter placement
Advance dilator over wire, then remove dilator	Determine guide wire in proper vessel before dilation	
Advance catheter over guide wire	Consider further dilation if difficulty encountered and consider appropriate depth of insertion for chosen site	
Remove guide wire	Determine that catheter is secure at all times	
Aspirate blood and flush each port with saline, cap/clamp	Identify problems with aspiration and determine if there is risk of embolism	Post-procedure verification
Secure catheter with suture/staples	Determine proper insertion distance and identify damage to catheter caused by needles or tools	
Place sterile dressing		
**Remove patient from Trendelenburg position**	Identify objects that may pull catheter during position change	
Order chest X-ray	Consider alternative verification methods	

Bold tasks are those added via the interview and survey process. All cognitive probes were found via the proposed methods. Cognitive probes are high-level reminders to be accompanied by more information as part of a CVC training curriculum

*TJC* the Joint Commission for the USA, *US* ultrasound

**Table 3:** Demographic information of SME participants

CTA phase	Department				Education			Sex		Age
	Internal medicine	Emergency medicine	Anaesthesiology	Nephrology	Resident	Fellow	Attending	Male	Female	Mean (SD) years
Simulation		1					1	1		*
Survey	7 (54 %)	3 (23 %)	3 (23 %)		3 (23 %)	5 (38 %)	5 (38 %)	12 (92 %)	1 (8 %)	35 (6.6)
Validation	2 (67 %)			1 (33 %)		1 (33 %)	2 (67 %)	2 (67 %)	1 (33 %)	42 (10.6)

Data are presented as number of participants (% of total)

* Age withheld to maintain anonymity of participant

The first procedure simulation and CTA-based interview required 2.5 hours to perform, while the verification procedures took less than 30 minutes per SME. Surveys were distributed in paper format, therefore time spent completing each survey could not be measured. This presents a total ‘scheduled’ time of 4 hours for SMEs, divided among four different physicians. The simulation scenario, CTA-based interview and survey were developed, performed, and transcribed by one researcher with the assistance of two students. Similar to other CTA methods, a review of published literature and discussions with SMEs were necessary to acquire a proper knowledge base prior to performing the CTA-based interview and creating the survey.

## Discussion

The goal of this project was to create a complete checklist for use in developing a training programme for first-year residents on the CVC procedure (internal jugular approach with ultrasound). To accomplish this goal in a timeframe suitable for the medical education field, an easy-to-implement modified cognitive task analysis method that makes use of an interview and surveys was developed. Using the simulation interview and survey data collected, a number of procedural steps not found within existing literature were identified, in addition to many cognitive decision points. These cognitive decision points have been composed as high-level reminders of the thought processes and decisions a medical professional makes while performing a CVC; the cognitive decision points will provide an outline of important teaching points from which a curriculum can be developed. As training material, the checklist could be provided to students accompanied by more information to enable them to confidently perform the procedure.

Clark and Estes [20] postulated that 30–35 hours of work by multiple CTA specialists is required to produce the knowledge content for one hour-long training session. While the time spent by researchers using the proposed method was on par with this estimate, execution of this modified task analysis method required minimal CTA training. Based on our experiences with traditional CTA methods, the amount of time required was greatly reduced; the number of PARI rehash sessions was reduced from five to one and part of the interview was simplified to a survey format. This creates the ability to perform an expanded task analysis on a number of linear medical procedures without overtaxing already overbooked medical professionals. These linear medical procedures (CVC, lumbar puncture, thoracentesis, paracentesis, etc.) are commonly taught to first-year residents. Using a modified CTA to develop the training curriculum for each procedure can ensure thorough instruction, practice, and assessment are completed before the procedure is attempted on a patient.

Although there are currently national guidelines for the sterile insertion of a CVC [1, 5, 28], there are no guidelines for the remaining steps. Every hospital or department will have its own preferred or recommended method based on current research and physician experience. For instance, some hospitals may recommend the subclavian approach for the CVC due to its reduced risk of CLABSI [28], while others may choose the internal jugular approach for its ease of insertion and ability to use ultrasound—which may make the internal jugular approach easier for inexperienced physicians. The Mayo Clinic strongly recommends the use of manometry to ensure venous placement of the catheter; [29] this particular step was not mentioned by any of the SMEs at the academic medical centre in this study. The differences in procedural steps between institutions highlight the need for individualized training programmes; resident physicians should be instructed in the procedural methods and equipment deemed appropriate by their hospital within national guidelines. This can be accomplished efficiently using the proposed task analysis technique.

The results in Table 2 should not be viewed as a standardized or all-encompassing guide to CVC insertion; they are a guide to create a training programme for the medical centre at which the study was conducted. The proposed task analysis method would enable any hospital to create a CTA-founded curriculum to train residents. Should changes in the procedural steps be recommended in the future, the CTA could also be quickly replicated by physicians—not necessarily experts in CTA—to reflect these modifications.

Current study limitations include the use of a partial-task training mannequin for analysis of a full procedure and the variations in teaching styles and procedure methods potentially present between different departments at the hospital. To address these concerns, participants were recruited from four different departments that all perform CVCs in their daily practice. This provides a broad base of knowledge and allows the creation of a more standardized training programme for the hospital as a whole. Another limitation of this study was the inability to measure the amount of time required for respondents to complete the printed surveys; however, the SMEs were allowed to complete the survey at their leisure and this activity did not remove them from patient care. A potential limitation was the use of only one SME interview. While three to four SMEs are generally preferred for a full CTA interview [9, 30], it has been shown that just one SME can provide significantly more information through a CTA interview process than four SMEs using simple free recall [16]. The interview provided by one SME, coupled with the ability of survey respondents to add their own procedural tasks, was found to be sufficient to gather an appropriate amount of information for this linear medical procedure.

## Conclusion

A modified cognitive task analysis using an adapted PARI methodology was used to create a list of 29 procedural tasks and 39 cognitive probes for the insertion of a CVC. This list provides the fundamental information necessary to develop the curriculum for a simulation-based training programme for first-year residents, with the goal of improving resident skills and knowledge of the procedure before they perform the procedure on a live patient. Using a standardized training method that incorporates the checklist and simulation, hospitals can enable their residents to receive proper training before being allowed to perform a procedure on a patient. Similar simulation-based training programmes have previously been shown to improve residents’ procedural skills and reduce the occurrence of complications associated with central line placement [6, 7, 13, 14].

The proposed modified task analysis technique has been shown to be effective in gathering a large amount of cognitive information in a short period of time requiring less scheduled time from highly trained personnel. Future work will employ this technique to develop similar checklists, curricula, and skills-assessment measures for other linear medical procedures. When the curricula have been created, a comparison to current training methods would be beneficial.

## Essentials

- Cognitive task analysis is an effective procedural training curriculum development method
- Cognitive task analysis can be time-consuming and overly complex for many procedures
- Our modified cognitive task analysis method can be used to quickly and thoroughly determine the procedural steps and cognitive aspects of linear medical procedures

## References

[1] The Joint Commission (2012). Preventing central line-associated bloodstream infections: a global challenge, a global perspective.

[2] Agency for Healthcare Research and Quality (2011). National healthcare quality report 2011.

[3] Berenholtz SM, Pronovost PJ, Lipsett PA, et al. Eliminating catheter-related bloodstream infections in the intensive care unit. Critical care medicine. Departments of Anesthesiology/CCM and Surgery, The Johns Hopkins University School of Medicine, Baltimore, MD, USA.; 2004;32(10):2014–20. 10.1097/01.ccm.0000142399.70913.2f 15483409

[4] Pronovost P, Needham D, Berenholtz S (2006). An intervention to decrease catheter-related bloodstream infections in the ICU. N Engl J Med..

[5] The Joint Commission (2012). National patient safety goals effective January 1, 2012.

[6] Britt RC, Novosel TJ, Britt LD, Sullivan M (2009). The impact of central line simulation before the ICU experience. Am J Surg.

[7] Evans LV, Dodge KL, Shah TD (2010). Simulation training in central venous catheter insertion: improved performance in clinical practice. Acad Med.

[8] Evans LV, Dodge KL (2010). Simulation and patient safety: evaluative checklists for central venous catheter insertion. Qual Saf Health Care.

[9] Yates K, Sullivan M, Clark R (2012). Integrated studies on the use of cognitive task analysis to capture surgical expertise for central venous catheter placement and open cricothyrotomy. Am J Surg.

[10] Evans LV, Morse JL, Hamann CJ, Osborne M, Lin Z, D’Onofrio G (2009). The development of an independent rater system to assess residents’ competence in invasive procedures. Acad Med.

[11] McGee DC, Gould MK (2003). Preventing complications of central venous catheterization. N Engl J Med..

[12] Dong Y, Suri HS, Cook DA (2010). Simulation-based objective assessment discerns clinical proficiency in central line placement: a construct validation. Chest.

[13] Barsuk JH, McGaghie WC, Cohen ER, Balachandran JS, Wayne DB (2009). Use of simulation-based mastery learning to improve the quality of central venous catheter placement in a medical intensive care unit. J Hosp Med..

[14] Velmahos GC, Toutouzas KG, Sillin LF (2004). Cognitive task analysis for teaching technical skills in an inanimate surgical skills laboratory. Am J Surg.

[15] Campbell J, Tirapelle L, Yates K (2011). The effectiveness of a cognitive task analysis informed curriculum to increase self-efficacy and improve performance for an open cricothyrotomy. J Surg Educ..

[16] Clark RE, Pugh CM, Yates K, Inaba K, Green DJ, Sullivan ME (2012). The use of cognitive task analysis to improve instructional descriptions of procedures. J Surg Res.

[17] Sullivan ME, Brown CV, Peyre SE (2007). The use of cognitive task analysis to improve the learning of percutaneous tracheostomy placement. Am J Surg.

[18] Sullivan ME, Ortega A, Wasserberg N, Kaufman H, Nyquist J, Clark R (2008). Assessing the teaching of procedural skills: can cognitive task analysis add to our traditional teaching methods?. Am J Surg.

[19] Procedures Consult. Central venous catheterization: internal jugular approach (internal medicine). http://www.proceduresconsult.com. Accessed 28 Sept 2012

[20] Clark RER, Estes F (1996). Cognitive task analysis for training. Int J Educ Res..

[21] DaRosa D, Rogers D, Williams R (2008). Impact of a structured skills laboratory curriculum on surgery residents’ intraoperative decision-making and technical skills. Acad Med.

[22] Klein G, Calderwood R (1989). Critical decision method for eliciting knowledge. IEEE Trans Syst Man Cybern..

[23] Bisantz A, Roth E (2007). Analysis of cognitive work. Rev Hum Factors Ergon..

[24] Roth EM (2008). Uncovering the requirements of cognitive work. Hum Factors..

[25] Hall EP, Gott SP, Pokorny RA. A procedural guide to cognitive task analysis: the PARI methodology. DTIC. 1995.

[26] Accreditation Council for Graduate Medical Education. RRC guidelines: guidelines for procedures and resuscitations (adopted by the RRC in September 2002).

[27] Woo M, Frank J, Lee A (2009). Effectiveness of a novel training program for emergency medicine residents in ultrasound-guided insertion of central venous catheters. CJEM..

[28] O’Grady N, Alexander M, Burns L (2011). Guidelines for the prevention of intravascular catheter-related infections. Clin Infect Dis.

[29] Mayo Foundation for Medical Education and Research. Clinical practice guideline—central venous cannulation. 2012.

[30] Smink DS, Peyre SE, Soybel DI, Tavakkolizadeh A, Vernon AH, Anastakis DJ (2012). Utilization of a cognitive task analysis for laparoscopic appendectomy to identify differentiated intraoperative teaching objectives. Am J Surg.

